# High-Conductivity, Flexible and Transparent PEDOT:PSS Electrodes for High Performance Semi-Transparent Supercapacitors

**DOI:** 10.3390/polym12020450

**Published:** 2020-02-14

**Authors:** Jiaxing Song, Guoqiang Ma, Fei Qin, Lin Hu, Bangwu Luo, Tiefeng Liu, Xinxing Yin, Zhen Su, Zhaobing Zeng, Youyu Jiang, Guannan Wang, Zaifang Li

**Affiliations:** 1 China-Australia Institute for Advanced Materials and Manufacturing (IAMM), Jiaxing University, Jiaxing 314001, China; songjx@zjxu.edu.cn (J.S.); hulin@zjxu.edu.cn (L.H.); xxyin@zjxu.edu.cn (X.Y.); suzhen@zjxu.edu.cn (Z.S.); zbzeng@zjxu.edu.cn (Z.Z.); 2 School of Applied Physics and Materials, Wuyi University, Jiangmen 529020, China; mgq1103@163.com; 3 Wuhan National Laboratory for Optoelectronics and School of Optical and Electronic Information, Huazhong University of Science and Technology, Wuhan 430074, China; by_qf@hust.edu.cn (F.Q.); tf_liu@hust.edu.cn (T.L.); youyujiang@hust.edu.cn (Y.J.); 4 College of Biomedical Engineering & the Key Laboratory for Medical Functional Nanomaterials, Jining Medical University, Jining 272067, China; chemwangguannan@126.com

**Keywords:** conducting polymer, PEDOT:PSS, high conductivity, semi-transparent supercapacitors, high power density

## Abstract

Herein, we report a flexible high-conductivity transparent electrode (denoted as S-PH1000), based on conducting polymer poly(3,4-ethylenedioxythiophene):poly(styrene sulfonate) (PEDOT:PSS), and itsapplication to flexible semi-transparentsupercapacitors. A high conductivity of 2673 S/cm was achieved for the S-PH1000 electrode on flexible plastic substrates via a H_2_SO_4_ treatment with an optimized concentration of 80 wt.%. This is among the top electrical conductivities of PEDOT:PSS films processed on flexible substrates. As for the electrochemical properties,a high specific capacitance of 161F/g was obtained from the S-PH1000 electrode at a current density of 1 A/g. Excitingly, a specific capacitance of 121 F/g was retained even when the current density increased to 100 A/g, which demonstrates the high-rate property of this electrode. Flexible semi-transparent supercapacitors based on these electrodes demonstrate high transparency, over 60%, at 550 nm. A high power density value, over 19,200 W/kg,and energy density, over 3.40 Wh/kg, was achieved. The semi-transparent flexible supercapacitor was successfully applied topower a light-emitting diode.

## 1. Introduction

Future electronic devices, such assupercapacitors, organic solar cells, wearable electronic devices, and mobile phones, are expected to be thin, light, transparent and flexible [1,2,3,4,5,6,7,8,9]. Among them, the supercapacitor is attracting increasing attention because of its high power density and short charging time [10,11,12,13,14,15]. Especially, semi-transparent or transparent flexible supercapacitors demonstrate more attractive futures due to their great potential as integrated power sources for displays and windows, such as buildings and aerospace vehicles [16,17,18]. Therefore, the development of semi-transparent or transparent flexible supercapacitors is of importance for future practical applications. However, developing semi-transparent or transparent flexible supercapacitors with a reasonable capacity, good charge/discharge ability and high power density is still a big challenge [19,20,21].

Having a transparent electrode is especially important for achieving high-performance, semi-transparent electronic devices. Among electrode materials [22,23,24,25], conducting polymer poly(3,4-ethylenedioxythiophene):poly(styrene sulfonate) (PEDOT:PSS) is a potential candidate due to its high transparency (over 90%), high conductivity (up to 10^3^ S/cm), good flexibility, electrochemical stability, and ease of processing by solution [26,27,28,29,30,31,32,33,34,35,36,37]. Recently, different kinds of PEDOT-based electrodes (such as nanofibrillar PEDOT, micrometer-thick PEDOT paper and free-standing PEDOT:PSS films) have been fabricated and applied successfully onto supercapacitors [38,39,40]. However, high-conductivity transparent solid-state flexible supercapacitors based on pure PEDOT materialshave rarely been covered [41,42,43]. Therefore, transparent PEDOT electrodes with high electrical conductivity arein urgent need in supercapacitors. Although dimethyl sulfoxide, ethylene glycol, surfactant or moderate acid treatment could enhance the conductivity of PEDOT:PSS, the obtained conductivity still cannot satisfy the needs ofhigh-performance electronic devices [33,34,35,36]. Concentrated sulfuric acid has demonstrated the ability to significantly enhance the conductivity of PEDOT:PSS [31,32]. However, the concentrated sulfuric acid is highly corrosive and easily damages the flexible plastic substrate. Lee et al. demonstrated the transfer fabrication of concentrated, sulfuric acid treated films from glass to flexible substrates [44]. Nevertheless, the transfer is based on the precise tuning of surface energy and interface adhesion, and the transfer procedure is complicated.

In this investigation, we report a flexible PEDOT:PSS electrode (denoted as S-PH1000) with a high conductivity of 2673 S/cm and treated withan optimized 80 wt.% H_2_SO_4_. This concentration of H_2_SO_4_ demonstrates much weaker corrosion than that of 98 wt.% and thus is compatible with plastic substrates (polyethersulfonate, PES). A high transparency (over 85%) and a sheet resistance of 89 Ohm/Sqwasobtained from the S-PH1000 electrode. Electrochemical measurement demonstrates a high specific capacitance of 161F/g, which is among the top values reported to date for PEDOT-based materials. Importantly, a specific capacitance of 121 F/g was maintained at a high current density, up to 100 A/g, suggesting a high-rate property of the S-PH1000 electrode. Further, symmetrical semi-transparent supercapacitors with a structure of PES/S-PH1000/H_3_PO_4_-PVA/S-PH1000/PES were successfully fabricated with a good transparency (over 60%) at the wavelength of 550 nm. The devices not only display remarkable electrochemical stability (over 10,000 cycles) and excellent flexible properties, but achieve a high power density of 19,200 W/kg and a comparable mass energy density of 3.40 Wh/kg. Finally, a light-emitting diode was demonstrated, powered by two semi-transparent supercapacitors in series. Our results indicate that the S-PH1000 electrode is a promising candidate for high performance, semi-transparent or transparent flexible energy devices.

## 2. Experimental Section

### 2.1. Preparation of the Flexible Highly-Conductive Transparent S-PH1000 Electrode

**Figure 1:**
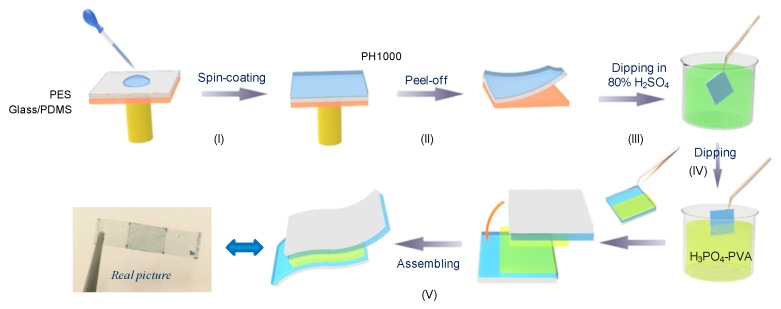
Schematic diagrams for the preparation of the S-PH1000 electrode and the flexible semi-transparent supercapacitor. Firstly, the PH1000 solution was spin-coated on PES substrate where the polyethersulfonate (PES) substrate was attached to a rigid glass substrate with a polydimethylsiloxane (PDMS) sheet in between. After spin coating, the PES/PH1000 film was peeled-off from the glass/PDMS substrate and heated on a hot plate. Then, the sample was immersed into an 80 wt.% H_2_SO_4_ solution at different temperatures to enhance the conductivity. After that, the S-PH1000 electrode was dipped into H_3_PO_4_-PVA glue and two pieces of electrodes were assembled to form a flexible semi-transparent supercapacitor. The last picture is the digital photograph of the semi-transparent flexible supercapacitor based on S-PH1000 films.

The detailed preparation of the S-PH1000 electrode is shown in Figure 1. Firstly, PES substrates (i-components) were adhered toglass substrates with polydimethylsiloxane (PDMS) sheets inbetween. Then, the PEDOT:PSS PH1000 aqueous solution (Heraeus, Hanau, Germany) was spin-coated on plasma-treated PES substrates. After that, the PES substrates with PH1000 films were peeled off the PDMS and dried at 150 °C on a hot plate for 10 min. The sulfuric acid treatment was performed by immersing the PES/S-PH1000 films in 80 wt.% H_2_SO_4_(diluted from a 98wt.% H_2_SO_4_) at different temperatures (25–110 °C). The films were taken out of the acid solutions and rinsed with deionized water three times. Finally, the films were dried at 120 °C for 5 min on a hot plate in air. The sheet resistance was measured by a four-point probe (RTS-8, Guangzhou, China) and the film thickness measurement was performed using a surface profiler (Veeco Dektak 150, Tucson, AZ, USA). The conductivity was calculated based on the sheet resistance and the film thickness. The transmittance (*T*) of PES/S-PH1000 films and supercapacitor devices wascharacterized by a UV-vis-NIR spectrophotometer (UV-3600, Shimadzu, Kyoto, Japan). A baseline correction was performed on the air for the transmittance.

### 2.2. Fabrication and Characterization of Semi-Transparent Supercapacitors

The detailed preparation procedure and pictures of the semi-transparent flexible supercapacitors are also included in Figure 1. The H_3_PO_4_-PVA gel electrolyte was prepared by mixing polyvinyl alcohol (PVA) (Mw = 130,000 g/mol; 98–99 mol% hydrolysed, Sigma-Aldrich, Saint Louis, MO, USA) powder (12 g), H_3_PO_4_ (12 g) and deionized water (120 mL) together. The mixture was heated to 85 °C with stirring until the solution became clear. Then, the solution was left standing for several hours and cooled to room temperature. Two pieces of the PES/S-PH1000 electrode were immersed into the PVA-H_3_PO_4_electrolyte for 5 min and then assembled into a supercapacitor by sandwiching a PVA-H_3_PO_4_ membrane as aseparator. Then, devices were kept in a fume hood to vaporize the excess water. The electrochemical performance was calculatedby gathering cyclic voltammetry (CV) and galvanostatic charging/discharging (GCD) measurements using an electrochemical workstation (CHI 660E, CH Instruments, Shanghai, China). The electrochemical impedance was tested from 1 mHz to 1 MHz with a potential amplitude of 100 mV (Autolab PGSTAT302N, Metrohm Autolab, Netherlands). The cycle life was determined by a battery test system (MTI). The specific capacitance is calculated according to the following equations:

*C* = *I*Δ*t*/Δ*E* (1)

*C_m_* = *C/m* = *I*Δ*t*/*m*Δ*E* (2)

where *C* is the total capacitance, *C_m_* is the specific capacitance, *I*is the discharge current, Δ*t* is the discharge time, Δ*E* is the potential window during the discharging process after the IR drop, and *m* is the weight of the active material. The mass energy density (*E*) and power density (*P*) play key roles in the practical application of supercapacitors and can be calculated as follows:

*E* = *C_m_*Δ*E*_0_^2^/2 (3)

*P* = *E*/Δ*t* (4)

where *C_m_* is the specific capacitance of the solid-state device, *I*is the discharge current, Δ*t* is the discharge time, Δ*E_0_*is obtained by a subtraction between the voltage window and the voltage drop.

## 3. Results and Discussion

### 3.1. Preparation and Characterization of the High Conductivity S-PH1000 Films

Figure 1 shows the schematic diagram of preparing the high-conductivity S-PH1000 electrodes and the semi-transparent supercapacitors. The key process to obtain high conductivity on flexible substrates is the optimization of the sulfuric acid (H_2_SO_4_) treatment. The oxidation and corrosion propertiesof the H_2_SO_4_ are strongly dependent on its concentration and processing temperature. Previously, we have demonstrated that flexible substrates (such as PES and PET) are quickly damaged in 98 wt.% H_2_SO_4_ when immersed in the solution [35].However, the H_2_SO_4_ treatment with a high concentration is beneficial to achieve high conductivity for PH1000 films. Here, we optimized the concentration of H_2_SO_4_ and the processing temperature to compromise the substrate safety and the conductivity of PH1000 films. We reduced the concentration of H_2_SO_4_ from 98 wt.% and observed that the substrates were intact when the concentration of the H_2_SO_4_ was reduced to 80 wt.%. Therefore, we employed the 80 wt.% H_2_SO_4_ to treat the PH1000 films on the PES substrates. Figure S1a demonstrates the conductivity of S-PH1000 films treated under temperatures ranging from 25 to 110 °C. It can be observed that the conductivity was enhanced gradually with the temperature increases. Notably, the PES substrates would not be damaged by 80 wt.% H_2_SO_4_ until the temperature increased to 120 °C. Figure S1b demonstrates the conductivity variation with dipping time, from which we can conclude that the optimal dipping time is 3 min. After being treated with 80 wt.% H_2_SO_4_ at 110 °C for 3 min, a high conductivity of 2673 S/cm was achieved from PH1000 films, which is among the top values reported to date, especially considering the flexible substrate.

**Figure 2:**
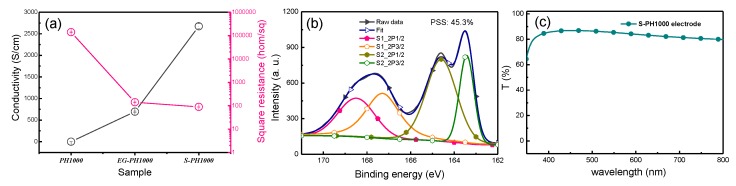
(**a**) Conductivity and square resistance of PH1000, EG-PH1000 and S-PH1000 films. (**b**) XPS spectrum of the S-PH1000 film. (**c**) The transmittance of S-PH1000 electrode.

Figure 2a demonstrates a comparison of the conductivity and the square resistance of pristine PH1000, 5 wt.% ethylene glycol-doped PH1000 (denote as EG-PH1000) and S-PH1000, from which we can find that S-PH1000 demonstrates the highest conductivity and the lowest square resistance. The XPS spectra of the S-PH1000 film are shown in Figure 2b, from which the PSS ratio was calculated to be 45.3%. This value is much lower than that of the pristine PH1000 of 73.8% [38]. The removal of PSS is beneficial to its air stability and conductivity [38], andthis is consistent with the high conductivity of 2673 S/cm. Besides, as shown in Figure 2c, the transmittance of the S-PH1000 electrode on PES substrate is over 85% at the wavelength of 550 nm, which demonstrates its high transparency.

### 3.2. Application of S-PH1000 Electrodes for Semi-Transparent Flexible Supercapacitors

**Figure 3:**
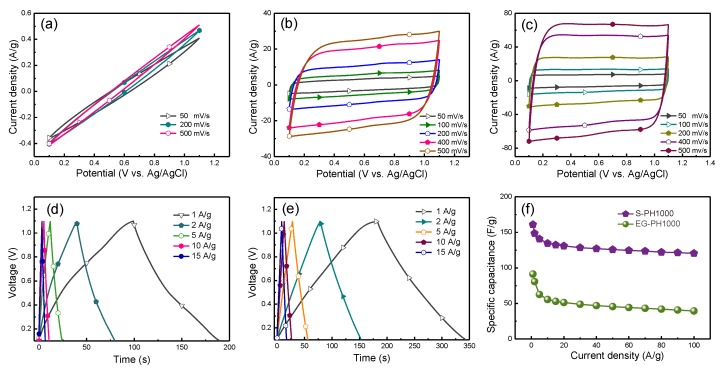
Cyclic voltammetry (CV) curves: (**a**) pristine PH1000 electrodes. (**b**) EG-PH1000 electrodes. (**c**) S-PH1000 electrodes. Charge-discharge profiles: (**d**) EG-PH1000 electrodes. (**e**) S-PH1000 electrodes. (**f**) A graphical representation of the specific capacitance as a function of the current density.

The electrochemical performance of PH1000, EG-PH1000 and S-PH1000 electrodes were characterized by cyclic voltammetry (CV) and galvanostatic charging/discharging (GCD) tests, utilizing a three-electrode configuration where pristine PH1000 and EG-PH1000 were used as reference samples. Figure 3a–c displays the CV curves of PH1000, EG-PH1000 and S-PH1000 film electrodes at scanning rates between 50 and 500 mV/s under a stable operation potential window between 0.1 and 1.1 V. The pristine PH1000 electrode is demonstrated to have aterrible electrochemical performance while the S-PH1000 electrode shows the best electrochemical properties among these electrodes, which is consistent with the square resistance values. The GCD curves of EG-PH1000 and S-PH1000 electrodes with a 1 V voltage window are shown in Figure 3de, respectively. Figure 3f displays a graphical representation of the specific capacitance of EG-PH1000 and S-PH1000 electrodes as a function of current density. Itcan be clearly observed that the S-PH1000 electrode displays a higher specific capacitance than that of the EG-PH1000 electrode. This could be caused by areduction in PSS that is not electrochemically active in the S-PH1000 electrode. Remarkably, a high specific capacitance of 161 F/g was obtained from the S-PH1000 electrode at a current density of 1 A/g, which is one of the highest values reported so far for PEDOT materials. More importantly, when the GCD current density was increased to 100 A/g, the S-PH1000 electrode maintained a high specific capacitance of 121 F/g, demonstrating the high-rate performance of the S-PH10000 electrode.

**Figure 4:**
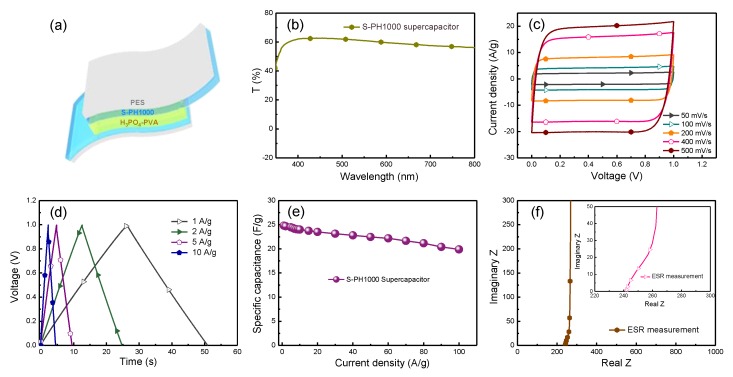
(**a**) Device structure of semi-transparent flexible supercapacitors: PES/S-PH1000/H_3_PO_4_-PVA/S-PH1000/PES. (**b**) The transmittance of the semi-transparent supercapacitor device. (**c**) CV curves of the S-PH1000 semi-transparent supercapacitors recorded at different scan rate of 50, 100, 200, 400 and 500 mV/s. (**d**) Galvanostatic charging/discharging (GCD) profiles of the semi-transparent supercapacitors recorded at different current densities of 1, 2, 5, 10 A/g. (**e**) A graphical representation of the variation of specific capacitance with respect to the current density. (**f**) Nyquist plot of the S-PH1000 semi-transparent flexible supercapacitor together with an enlarged photograph in the inset.

Considering its high conductivity over 2673 S/cm, transmittance over 85% and flexibility, the S-PH1000 electrodes are applied to fabricate high-performance, symmetric, semi-transparent, flexible supercapacitors (Figure 4a). Figure 4b displays the transmittance of the S-PH1000-based semi-transparent supercapacitor. Consequently, a transmittance of over 60% can be achieved for the overall supercapacitor. Figure 4c shows the CV characteristics of the semi-transparent, flexible supercapacitors based on S-PH1000 electrodes. The rectangular feature of the CV curves indicatesthat there is excellent electrical conductivity for the S-PH1000-based devices. The GCD profiles of S-PH1000 semi-transparent flexible supercapacitors are shown in Figure 4d. The specific capacitance is calculated to be 24.8 F/g under a GCD current density of 1A/g. Figure 4e displays a graphical representation of the variation of the device’s specific capacitance with respect to the current density, and it turns out that there is a high-rate property in the semi-transparent supercapacitors. Further, the conductivity of the S-PH1000 supercapacitors was characterized by the electrochemical impedance spectroscopy measurement (Figure 4f), which shows a resistance of 242 ohm that is in accordance with the square resistance of the S-PH1000 electrodes.

**Figure 5:**
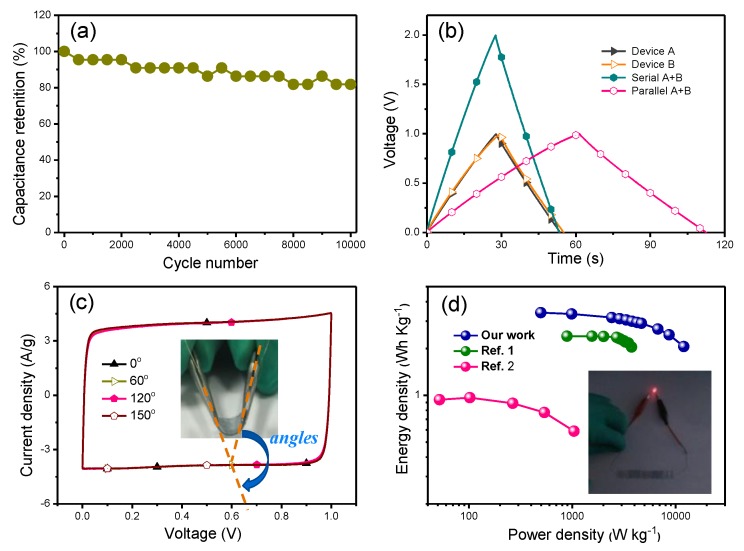
(**a**) Cycling stability of the S-PH1000 supercapacitor at a high scan rate of 100 mV/s. (**b**) GCD profiles of independent, series and parallel S-PH1000 electrodes based on supercapacitors of device A and B at a charge current density of 1 A/g. (**c**) CV curves of the semi-transparent flexible supercapacitors under different bending angles (60°, 120° and 150°). (**d**) Energy density and power density Ragone plots of the PEDOT-based supercapacitors in our work and reported in the literatures. Ref. 1 denotes the reference of *ACS Nano*, 2014, 8, 1500 while Ref. 2 denotes *Energy Environ. Sci.* 2015, 8, 1339. The inset is the demonstration of the supercapacitors driving a light-emitting diode.

In addition, a long-term cycle stability was performed at a high scan rate 100 mV/s (Figure 5a) which showed that more than 80% specific capacitance was maintained after 10,000 cycles,indicating the excellent electrochemical stability of the S-PH1000-based devices. The initial reduction in capacitance should be attributed to the loss of water from the H_3_PO_4_-PVA gel electrolyte, resulting from the heat generated during cycles. The series and parallel semi-transparent flexible supercapacitors were fabricated and investigated as well. Figure 5b displays the GCD profiles of these semi-transparent flexible supercapacitors derived from devices A and B at a current density of 1 A/g. It can be observed that the devices in both series and parallel could double the performance. Besides, the mechanical stability of the device under conditions of various bending angles (60°, 120° and 150°) was performed and demonstrated the good stability of fabricated supercapacitors by CV test verification (Figure 5c). As energy and power densities play important roles for practical applications, the values of these metrics were further calculated according to Formulas (3) and (4). Figure 5d shows the plot of the energy density and power density of the PEDOT-based supercapacitors in our work and reported in the literature [39,40], from which we can find that the supercapacitors in our work can achieve a relatively better device performance. Because of its high-power density and comparable energy density, two semi-transparent supercapacitor devices in series were successfully applied to drive the light-emitting diode (see inset, Figure 5d). All above results have demonstrated that the S-PH1000 film is a good candidate as an efficient transparent flexible electrode for the supercapacitors.

## 4. Conclusions

High performance flexible semi-transparent supercapacitors based on high-conductivity conducting polymer PEDOT:PSS electrodes (2673 S/cm) has been reported by optimizing the concentration of sulfuric acid and treated temperature. The resulting S-PH1000 electrode demonstrates a high specific capacitance of 161 F/g at a current density of 1 A/g and maintains a high value of 121 F/g at 100 A/g, ensuring its high-rate property. Lastly, flexible semi-transparent supercapacitors based on S-PH1000 electrodes deliver a high-powerdensity over 19,200 W/kg and a high energy density of 3.40 Wh/kg with a high transparency of over 60%. In addition to supercapacitors, this flexible, transparent electrode is also expected to be applied to other electronic devices (such as organic solar cells and thermoelectric) due to its high electrical conductivity, transparency and excellent flexibility.

## Supplementary Materials

The following are available online at https://www.mdpi.com/2073-4360/12/2/450/s1, Figure S1: (a) The conductivity values of S-PH1000 electrodes treated by 80 wt.% H_2_SO_4_ under different temperatures from 25 to 110 °C, (b) the conductivity values of S-PH1000 electrodes treated by 80 wt.% H_2_SO_4_ under different time., Table S1: Data for semi-transparent supercapacitor based on S-PH1000 electrode, Table S2: Data for semi-transparent supercapacitor device A and B in separated, series and parallel at a discharge current density of 1 A/g.

## References

[1] Wu H., Kong D., Ruan Z., Hsu P.-C., Wang S., Yu Z., Carney T.J., Hu L., Fan S., Cui Y. (2013). A transparent electrode based on a metal nanotrough network. Nat. Nanotechnol..

[2] Donolato M., Tollan C., Porro J.M., Berger A., Vavassori P. (2013). Flexible and stretchable polymers with embedded magnetic nanostructures. Adv. Mater..

[3] Gwon H., Hong J., Kim H., Seo D.-H., Jeon S., Kang K. (2014). Recent progress on flexible lithium rechargeable batteries. Energy Environ. Sci..

[4] Yu W.J., Lee S.Y., Chae S.H., Perello D., Han G.H., Yun M., Lee Y.H. (2011). Small hysteresis nanocarbon-based integrated circuits on flexible and transparent plastic substrate. Nano Lett..

[5] Chen T., Hao R., Peng H., Dai L. (2015). High-performance, stretchable, wire-shaped supercapacitors. Angew. Chem..

[6] Guan X., Kong D., Huang Q., Cao L., Zhang P., Lin H., Lin Z., Yuan H. (2019). In situ growth of a high-performance all-solid-state electrode for flexible supercapacitors based on a PANI/CNT/EVA composite. Polymers.

[7] Chen Y., Chen T., Dai L. (2015). Layer-by-layer growth of CH(3)NH(3)PbI(3-x)Clx for highly efficient planar heterojunction perovskite solar cells. Adv. Mater..

[8] Xiao X., Peng X., Jin H., Li T., Zhang C., Gao B., Hu B., Huo K., Zhou J. (2013). Freestanding mesoporous VN/CNT hybrid electrodes for flexible all-solid-state supercapacitors. Adv. Mater..

[9] Ding Z., Hao Z., Meng B., Xie Z., Liu J., Dai L. (2015). Few-layered graphene quantum dots as efficient hole-extraction layer for high-performance polymer solar cells. Nano Energy.

[10] Hu Z., Xiao X., Chen C., Li T., Huang L., Zhang C., Su J., Miao L., Jiang J., Zhang Y. (2015). Al-doped α-MnO_2_ for high mass-loading pseudocapacitor with excellent cycling stability. Nano Energy.

[11] Cheng Y., Huang L., Xiao X., Yao B., Yuan L., Li T., Hu Z., Wang B., Wan J., Zhou J. (2015). Flexible and cross-linked N-doped carbon nanofiber network for high performance freestanding supercapacitor electrode. Nano Energy.

[12] Hui J., Wei D., Chen J., Yang Z. (2019). Polyaniline nanotubes/carbon cloth composite electrode by thermal acid doping for high-performance supercapacitors. Polymers.

[13] Wang J., Li X., Zi Y., Wang S., Li Z., Zheng L., Yi F., Li S., Wang Z.L. (2015). A Flexible Fiber-Based Supercapacitor-Triboelectric-Nanogenerator Power System for Wearable Electronics. Adv. Mater..

[14] Zhou Y., Xu H., Lachman N., Ghaffari M., Wu S., Liu Y., Ugur A., Gleason K.K., Wardle B.L., Zhang Q.M. (2014). Advanced asymmetric supercapacitor based on conducting polymer and aligned carbon nanotubes with controlled nanomorphology. Nano Energy.

[15] Yuan L., Xiao X., Ding T., Zhong J., Zhang X., Shen Y., Hu B., Huang Y., Zhou J., Wang Z.L. (2012). Paper-based supercapacitors for self-powered nanosystems. Angew. Chem..

[16] Niu Z., Zhou W., Chen J., Feng G., Li H., Hu Y., Ma W., Dong H., Li J., Xie S. (2013). A repeated halving approach to fabricate ultrathin single-walled carbon nanotube films for transparent supercapacitors. Small.

[17] Jung H.Y., Karimi M.B., Hahm M.G., Ajayan P.M., Jung Y.J. (2012). Transparent, flexible supercapacitors from nano-engineered carbon films. Sci. Rep..

[18] Chen T., Peng H., Durstock M., Dai L. (2014). High-performance transparent and stretchable all-solid supercapacitors based on highly aligned carbon nanotube sheets. Sci. Rep..

[19] Hiralal P., Wang H., Unalan H.E., Liu Y., Rouvala M., Wei D., Andrew P., Amaratunga G.A.J. (2011). Enhanced supercapacitors from hierarchical carbon nanotube and nanohorn architectures. J. Mater. Chem..

[20] Li H., Zhao Q., Wang W., Dong H., Xu D., Zou G., Duan H., Yu D. (2013). Novel planar-structure electrochemical devices for highly flexible semitransparent power generation/storage sources. Nano Lett..

[21] Rodriguez-Moreno J., Navarrete-Astorga E., Dalchiele E.A., Schrebler R., Ramos-Barrado J.R., Martin F. (2014). Vertically aligned ZnO@CuS@PEDOTcore@shellnanorod arrays decorated with MnO(2) nanoparticles for a high-performance and semi-transparent supercapacitor electrode. Chem. Commun..

[22] Han H., Lee J.S., Cho S. (2019). Comparative studies on two-electrode symmetric supercapacitors based on polypyrrole: Poly(4-styrenesulfonate) with different molecular weights of poly(4-styrenesulfonate). Polyme..

[23] Wang G., Zhang L., Zhang J. (2012). A review of electrode materials for electrochemical supercapacitors. Chem. Soc. Rev..

[24] Fang Y., Luo B., Jia Y., Li X., Wang B., Song Q., Kang F., Zhi L. (2012). Renewing functionalized graphene as electrodes for high-performance supercapacitors. Adv. Mater..

[25] Gul H., Shah A.-U.-H.A., Bilal S. (2019). Achieving ultrahigh cycling stability and extended potential window for supercapacitors through asymmetric combination of conductive polymer nanocomposite and activated carbon. Polymers.

[26] Hu L., Song J., Yin X., Su Z., Li Z. (2020). Research progress on polymer solar cells based on PEDOT:PSS electrodes. Polymers.

[27] Kim N., Lee B.H., Choi D., Kim G., Kim H., Kim J.R., Lee J., Kahng Y.H., Lee K. (2012). Role of interchain coupling in the metallic state of conducting polymers. Phys. Rev. Lett..

[28] Li Z., Sun H., Hsiao C.-L., Yao Y., Xiao Y., Shahi M., Jin Y., Cruce A., Liu X., Jiang Y. (2018). A free-standing high-output power density thermoelectric device based on structure-ordered PEDOT: PSS. Adv. Electron. Mater..

[29] Li Z., Qin F., Liu T., Ge R., Meng W., Tong J., Xiong S., Zhou Y. (2015). Optical properties and conductivity of PEDOT: PSS films treated by polyethylenimine solution for organic solar cells. Org. Electron..

[30] Fabretto M.V., Evans D.R., Mueller M., Zuber K., Hojati-Talemi P., Short R.D., Wallace G.G., Murphy P.J. (2012). Polymeric Material with Metal-Like Conductivity for Next Generation Organic Electronic Devices. Chem. Mater..

[31] Xia Y., Sun K., Ouyang J. (2012). Solution-processed metallic conducting polymer films as transparent electrode of optoelectronic devices. Adv. Mater..

[32] Kim N., Kee S., Lee S.H., Lee B.H., Kahng Y.H., Jo Y.R., Kim B.J., Lee K. (2014). Highly conductive PEDOT:PSSnanofibrils induced by solution-processed crystallization. Adv. Mater..

[33] Ouyang J. (2013). “Secondary doping” methods to significantly enhance the conductivity of PEDOT: PSS for its application as transparent electrode of optoelectronic devices. Displays.

[34] Li Z., Meng W., Tong J., Zhao C., Qin F., Jiang F., Xiong S., Zeng S., Xu L., Hu B. (2015). A nonionic surfactant simultaneously enhancing wetting property and electrical conductivity of PEDOT:PSS for vacuum-free organic solar cells. Sol. Energy Mater. Sol. Cells.

[35] Meng W., Ge R., Li Z., Tong J., Liu T., Zhao Q., Xiong S., Jiang F., Mao L., Zhou Y. (2015). Conductivity Enhancement of PEDOT:PSS Films via Phosphoric Acid Treatment for Flexible All-Plastic Solar Cells. ACS Appl. Mater. Inter..

[36] Rwei S.-P., Lee Y.-H., Shiu J.-W., Sasikumar R., Shyr U.-T. (2019). Characterization of solvent-treated PEDOT:PSS thin films with enhanced conductivities. Polymers.

[37] Morris J.D., Wong K.M., Penaherrera C.D., Payne C.K. (2016). Mechanism of the biomolecular synthesis of PEDOT:PSS: Importance of heme degradation by hydrogen peroxide. Biomater. Sci..

[38] Li Z., Ma G., Ge R., Qin F., Dong X., Meng W., Liu T., Tong J., Jiang F., Zhou Y. (2016). Free-Standing Conducting Polymer Films for High-Performance Energy Devices. Angew. Chem..

[39] D’Arcy J.M., El-Kady M.F., Khine P.P., Zhang L., Lee S.H., Davis N.R., Liu D.S., Yeung M.T., Kim S.Y., Turner C.L. (2014). Vapor-phase polymerization of nanofibrillar poly (3, 4-ethylenedioxythiophene) for supercapacitors. ACS Nano.

[40] Anothumakkool B., Soni R., Bhange S.N., Kurungot S. (2015). Novel scalable synthesis of highly conducting and robust PEDOT paper for a high performance flexible solid supercapacitor. Energy Environ. Sci..

[41] Zhang C., Higgins T.M., Park S.-H., O’Brien S.E., Long D., Coleman J.N., Nicolosi V. (2016). Highly flexible and transparent solid-state supercapacitors based on RuO2/PEDOT:PSS conductive ultrathin films. Nano Energy.

[42] Cheng T., Zhang Y.-Z., Zhang J.-D., Lai W.-Y., Huang W. (2016). High-performance free-standing PEDOT:PSS electrodes for flexible and transparent all-solid-state supercapacitors. J. Mater. Chem. A.

[43] Cai G., Darmawan P., Cui M., Wang J., Chen J., Magdassi S., Lee P.S. (2016). Highly Stable Transparent Conductive Silver Grid/PEDOT:PSS Electrodes for Integrated Bifunctional Flexible Electrochromic Supercapacitors. Adv. Energy Mater..

[44] Kim N., Kang H., Lee J.H., Kee S., Lee S.H., Lee K. (2015). Highly conductive all-plastic electrodes fabricated using a novel chemically controlled transfer-printing method. Adv. Mater..

