# Supplementary material for: High-Conductivity, Flexible and Transparent PEDOT:PSS Electrodes for High Performance Semi-Transparent Supercapacitors
Source: Polymers (Basel). 2020 Feb 14;12(2):450. doi: 10.3390/polym12020450 (PMC7077632; doi:10.3390/polym12020450)
Supplement: Supplementary file 1 [file polymers-12-00450-s001.pdf]

Supporting Information

High-Conductivity, Flexible and Transparent PEDOT:PSS Electrodes for High Performance Semi-Transparent Supercapacitors

Jiaxing Song [1,†], Guoqiang Ma [2,†], Fei Qin [3], Lin Hu [1], Bangwu Luo [3], Tiefeng Liu [3], Xinxing Yin [1], Zhen Su [1], Zhaobing Zeng [1], Youyu Jiang [3], Guannan Wang [4] and Zaifang Li [1,*]

[1] China-Australia Institute for Advanced Materials and Manufacturing (IAMM), Jiaxing University, Jiaxing 314001, China; songjx@zjxu.edu.cn (J.S.); hulin@zjxu.edu.cn (L.H.); xxyin@zjxu.edu.cn (X.Y.); suzhen@zjxu.edu.cn (Z.S.); zbzeng@zjxu.edu.cn (Z.Z.)

[2] School of Applied Physics and Materials, Wuyi University, Jiangmen 529020, China; mgq1103@163.com

[3] Wuhan National Laboratory for Optoelectronics and School of Optical and Electronic Information, Huazhong University of Science and Technology, Wuhan 430074, China; by_qf@hust.edu.cn (F.Q.); luoxiaowu870413@163.com (B.L.); tf_liu@hust.edu.cn (T.L.); youyujiang@hust.edu.cn (Y.J.)

[4] College of Biomedical Engineering & the Key Laboratory for Medical Functional Nanomaterials, Jining Medical University, Jining 272067, China; chemwangguannan@126.com

* Correspondence: zaifang.li@zjxu.edu.cn

† These authors contributed equally to this work.

Table S1. Data for semi-transparent supercapacitor based on S-PH1000 electrode.

Current density (A/g)	Time (s)	Voltage (V)	Cv (F/g)	E (W h/kg)	P (W/kg)
1	24.64	0.993	24.8	3.40	497
2	12.17	0.986	24.7	3.33	986
5	4.72	0.965	24.5	3.16	2413
6	3.89	0.959	24.3	3.11	2877
7	3.29	0.953	24.2	3.05	3335
8	2.846	0.946	24.1	2.99	3784
9	2.505	0.939	24.0	2.94	4226
10	2.24	0.933	24.0	2.90	4665
15	1.423	0.899	23.7	2.67	6743
20	1.018	0.866	23.5	2.45	8660
30	0.617	0.8	23.1	2.06	12000
40	0.419	0.735	22.8	1.71	14700
50	0.301	0.67	22.5	1.40	16750
60	0.224	0.606	22.2	1.13	18180
70	0.168	0.543	21.7	0.89	19005
80	0.127	0.48	21.2	0.68	19200
90	0.095	0.419	20.4	0.50	18855
100	0.071	0.357	19.9	0.35	17850

Table S2. Data for semi-transparent supercapacitor device A and B in separated, series and parallel at a discharge current density of 1 A/g.

Device (1 A /g)	Time (s)	Voltage (V)	Cv (F/g)	E (Wh kg^{-1})	P (W Kg^{-1})
A	25.8	0.9934	26	3.61	503
B	25.6	0.9922	25.8	3.58	503
A+B (series)	25.6	1.986	12.9	7.17	1008
A+B (parallel)	51.1	0.9962	51.3	7.13	502

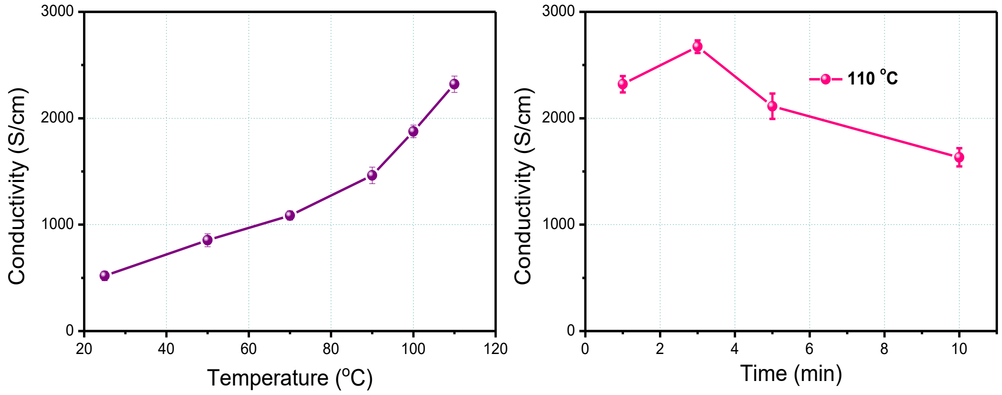

Figure S1. (**a**) The conductivity values of S-PH1000 electrodes treated by 80 wt.% H_2SO_4 under different temperatures from 25 to 110 °C, (**b**) the conductivity values of S-PH1000 electrodes treated by 80 wt.% H_2SO_4 under different time.
